# Measurement of Disease Activity in Ecuadorian Patients with Rheumatoid Arthritis: Does RAPID3 Correlate with Traditional Indexes?

**DOI:** 10.1155/2019/6940401

**Published:** 2019-03-20

**Authors:** María Fernanda Zurita, Adriana Iglesias, Emanuel Vanegas, Adriana Luzuriaga, Luis Zurita

**Affiliations:** ^1^Universidad Espíritu Santo, School of Medicine, Samborondón, Ecuador; ^2^Department of Rheumatology, Unidad de Enfermedades Reumáticas y Autoinmunes [UNERA] (Unit of Rheumatology and Autoimmune Diseases), Guayaquil, Ecuador

## Abstract

**Objective:**

The aim of this study is to demonstrate if routine assessment of patient index data 3 has a correlation with disease's activity as much as disease activity score 28, clinical disease activity index, and simplified disease activity index in Ecuadorian patients with rheumatoid arthritis seen in Unidad de Enfermedades Reumáticas y Autoinmunes [UNERA] from December 2016 to December 2017.

**Methods:**

This is a retrospective study in 200 patients that fulfill the American College of Rheumatology 2010 criteria for diagnosis of rheumatoid arthritis. The patients were evaluated from December 2016 to December 2017. Descriptive analyses were carried out, also Pearson correlation was used, and, to give a better clinical significance, a chi-square test was conducted. Whenever assumptions of chi-square test were violated, a Fisher's exact test was reported.

**Results:**

RAPID3 correlated best with DAS28 (*r*.83, p < 0.001), followed by CDAI (*r*.80, p < 0.001) and then SDAI (*r*.77, p < 0.001).

**Conclusion:**

RAPID3 is a questionnaire that only takes 10 seconds to calculate and correlates in a significant way with traditional clinical measures that require more time to perform, saving time in busy health facilities.

## 1. Introduction

Rheumatoid arthritis is a chronic, systemic autoimmune disease characterized by a symmetrical inflammation of synovial joints. Etiology is still unknown, although genetics and environmental factors have been shown to increase susceptibility to the disease [1]. This pathology affects one percent of the population and is more common in women between their 40 and 70 decades of life [2]. Nowadays, the management of rheumatoid arthritis is based on treating to target, a strategy that mainly attempts to attain remission or the lowest disease's activity possible in case that remission is not accomplished [3]. This is best achieved by measuring the patient's disease activity with indexes which allow physicians to take the best therapeutic decisions regarding each patient and establishing a prognosis. In the last decades, several instruments have been used in order to achieve this purpose; they have been validated worldwide and are trustworthy.

Among them, clinical indexes that combine physical examination, questionnaires, inflammatory markers, or diagnostics images aim to create scores that grant physicians the ability to take decisions based on reproducible measures. These measures should be able to reach an objective assessment about the disease's activity degree, the functional impact, and the patient's quality of life in different stages of its illness [4]. All together, these measures are known as clinimetrics.

There has also been a lot of interest in the evaluation of the disease from the patient's own perspective, which has led to creating the patient-reported outcomes (PRO) [5]. PRO is defined as the patients' use of tools that allow them to make an objective evaluation of their disease in order to take the right clinical and therapeutic decisions [6].

Traditionally, the indexes used to measure the disease's activity are the disease activity score 28 (DAS28) and the clinical disease activity index (CDAI) or simplified disease activity index (SDAI), which include the counting of painful and inflamed joints and visual analog scale (VAS) performed by the doctor and patient (time needed to complete is approximately 2 minutes) [7]. DAS28 and SDAI also incorporate laboratory tests like the erythrocyte sedimentation rate (ESR) and C-reactive protein level (CRP), respectively [8].

In addition, a more practical index that does not include the counting of inflamed joints or blood tests was also introduced. The routine assessment of patient index data 3 (RAPID3) measures pain, functional impairment, and patient's global estimate in a 10 to 0 score, in which 10 is the worst possible situation and 0 is the best [9]. It is a questionnaire filled by the patient at the doctor's office, and it only takes 10 seconds to calculate [10].

The purpose of our study is to assess whether RAPID3 has a correlation with disease's activity as much as DAS-28, CDAI, and SDAI in a population of Ecuadorian patients with rheumatoid arthritis seen in Unidad de Enfermedades Reumáticas y Autoinmunes [UNERA] from December 2016 to December 2017.

## 2. Patients and Methods

### 2.1. Patients

We carried out a retrospective study involving 200 patients diagnosed with rheumatoid arthritis from December 2016 to December 2017 at Unidad de Enfermedades Reumáticas y Autoinmunes [UNERA] (Unit of Rheumatology and Autoimmune Diseases) located in Guayaquil, Ecuador. All patients were at least 18 years old. Demographic and clinical variables such as age, sex, and body mass index (BMI), years with the disease, laboratory tests, medications, and comorbidities were collected using medical records from the institution. If the patients did not have laboratory results, we considered it as missing data and they were not included in our database. An expert rheumatologist carefully estimated the formal joint count. In addition, we also collected data about the treatment that the patients were receiving, which included conventional and synthetics disease modifying antirheumatic drugs (DMARDs), glucocorticoids, biologics, and JAK-1 and JAK- 3 inhibitors (tofacitinib).

Diagnosis of rheumatoid arthritis was made following the 2010 American College of Rheumatology/European League Against Rheumatism Classification criteria [11]. Moreover, activity of disease was assessed by 4 methods, namely, RAPID3, DAS28, CDAI, and SDAI.

DAS28 is an index that measures the degree of activity in a patient by counting 28 joints such as shoulders, elbows, wrists, metacarpophalangeal joints, proximal interphalangeal joints, knees, and ankles. It also includes the erythrocyte sedimentation rate (ESR) with a maximum of 200 and the visual analog scale of 100. Using the VAS, the patients qualify how the disease have affected them in general the week prior to the evaluation, giving a score of 100 in the worst situation and 0 in the best. The values that we considered were remission <2.6, low activity between 2.61 and 3.2, moderate activity> 3.2 to 5.1, and high activity >5.2 [12].

CDAI takes into account the patient global assessment of disease activity (PGA), which is a measure with a visual analog scale of over 10 that is completed by the patient according to how its illness has affected him/her in the week prior to its consult. It also includes the evaluator's global disease activity (EGA) from 0 to 10, which an evaluator assesses according to how he sees the patient in the last week [13]. The counting of painful and inflamed joints on 28 is also studied. The values of CDAI that we took in consideration were remission from 0 to 2.8; low activity: between 2.9 and 10; moderate activity: 10.1 to 22; and high activity: 22.1 to 76.

SDAI includes the counting of tender and swollen joints on 28, PGA and physician global assessment that are measures in a scale from 0 to 10, and the C-reactive protein (CRP) level (0.1-10 mg/dl). The values of SDAI were remission ≤3.3, low activity ≤11, moderate activity ≤26, and high activity >26 [14].

RAPID3 is a questionnaire that is filled by the patient; it consists of 10 questions about their daily activities that are rated on a maximum of 10; visual analog scale of pain that the patient has felt in the last week, over 10; and visual analog scale on how the disease has affected him or her in general the week prior to evaluation that is also scored on a scale of 10 [15]. The values of RAPID3 that we included were remission 0-3, low activity 3.1 to 6, moderate activity 6.1 to 12, and high activity > 12.

These methods were analyzed both quantitatively as continuous variables (score) and clinically as categorical variables (remission, mild, moderate, and high).

### 2.2. Sample Size

We used G*∗*Power Ver. 3.0.10 to calculate the sample size for a chi-square test for association. Setting a power of 0.8 to detect a medium size effect (0.3) with 9 degrees of freedom and an *α* error probability of 0.05, total sample size was 174. However, we included 200 patients to increase power and overcome type II error in anticipation of missing data, outliers, or nonnormality. Actual power with 200 participants was 0.86.

### 2.3. Statistical Analysis

Descriptive analyses (frequency, percentage, and standard deviation) were carried out for demographic and clinical variables. The Pearson product-moment correlation was used to ascertain the strength and direction of a relationship between the scores of each method used to assess the activity of RA. For such purposes, the scores were used as continuous variables. To give a better clinical significance, a chi-square test for association was conducted between the same methods used to assess the activity of disease, but for these analyses the variables were treated as categorical. Whenever assumptions of chi-square test were violated, a Fisher's exact test was reported.

Logistic regressions were adjusted for age, gender, years with rheumatoid arthritis, and disease activity assessed by DAS28. All statistical analyses were carried out using SPSS v24.0 (IBM, Armonk, NY, USA).* P* < 0.05 was considered significant.

## 3. Results

Among the 200 patients studied, 85.5% were female and 14.5% were male, with the age ranging from 19 to 79 (mean, 55.4; SD, 13.5) years. The mean duration of suffering from rheumatoid arthritis was 8.8 (SD 7.0) years; 166 patients were seropositive and 34 were seronegative. Regarding comorbidities 27.5% had arterial hypertension, 14.5% had type 2 diabetes mellitus, 15% had hyperlipidemia, and 42% osteoporosis; the standard deviation of BMI was 4.5. In regard to the treatment, 98% were taking conventional DMARDs, 36% biologics, 82.5% corticosteroids, and 8% tofacitinib (Table 1).

### 3.1. Disease Activity

Mean scores were 14.1 for RAPID3, 4.9 for DAS28, 21.6 for CDAI, and 23.7 for SDAI. The highest proportions of patients were consistent with a highly active disease (IV) regardless of the method used. For further details, Table 2 best describes the proportions of the subgroups of disease activity and their averages according to each method.

High activity according to DAS28 was presented in 49.5% of the patients, 78.9% of whom also had high activity score according to RAPID3. Regarding moderate activity, 33.5% of patients were identified in DAS28 score, and 71.4% with RAPID3. 3% of patients showed low activity with DAS score and 11.11% had low activity with RAPID3. Regarding remission, 14% were in remission with DAS28 and 91.3% with RAPID3.

Concerning CDAI, 43.5% had high activity and 70.1% of those showed high activity with RAPID3. Regarding moderate activity, 29.5% of patients were identified as having moderate activity, with CDAI and RAPID3 identifying 57.1% of those patients.

17.5% of patients had low activity with CDAI and 83.3% had low activity with RAPID3, respectively. 9.5% of patients were on remission according to CDAI and 73.9% of those were in remission according to RAPID3.

According to the results related to SDAI, 39% of patients showed high activity, of whom 63.2% were identified as having high activity with RAPID3. Moderate activity was seen in 37% of patients with SDAI and 66.7% of patients with RAPID3. 13,5% of patients were identified as having low activity with SDAI and 55,6% of those were identified with RAPID3, respectively. Finally remission was seen in 10.5% of patients with SDAI and 73.9% with RAPID3 (Table 3).

### 3.2. Correlations

**Figure 1:**
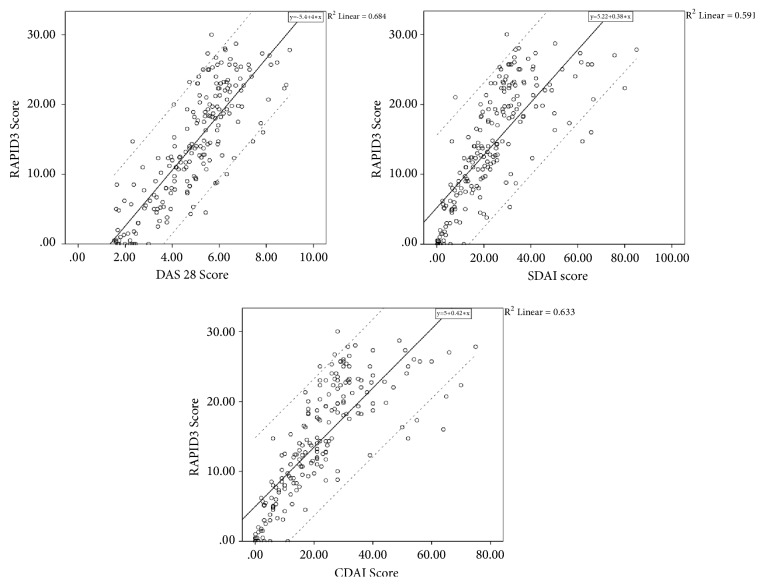
Correlation between RAPID3 and other rheumatoid arthritis scores.

RAPID3 correlated best with DAS28 (*r*.83, p < 0.001), followed by CDAI (*r*.80, p < 0.001) and then SDAI (*r*.77, p < 0.001) (Figure 1). Chi-square tests for associations were statistically significant between RAPID3 and the other methods (p < 0.001). Cramer's V showed a moderately high association between RAPID3 and CDAI (*φ*=.634), while association between RAPID3 and DAS28 (*φ*=.590) and SDAI (*φ*=.583) was moderate (Table 3).

## 4. Discussion

The first disease activity index was developed in 1950. Since then, several others have been created in order to measure the disease activity in patients with rheumatoid arthritis. Clinical indexes offer the best option for physicians so they can be able to achieve the treat to target strategy and give this disease an efficient treatment.

The present study found a lineal relation between all the clinical measures and RAPID3. DAS-28 had the strongest correlation (r.83, p < 0.001), followed by CDAI (r.80, p < 0.001) and then SDAI (r.77, p < 0.001), demonstrating that RAPID3 is a congruent measure.

Ballesteros et al. studied a population of 119 Colombian patients that showed similar results to the ones we presented. However, they found a stronger correlation between RAPID3 with SDAI (r 0.75, p < 0.001) and CDAI (r 0.75, p < 0.001). Our study suggested that there is a better correlation between RAPID-3 and DAS-28 [16]. Moreover, another publication made by Bossert et al. also demonstrated comparable results between RAPID3 and DAS-28 r_s_ (0.637), CDAI r_s_ (0.713) and SDAI rs (0.714), respectively [17].

From a clinical standpoint, our study indicated that RAPID3 had the best correlation with CDAI (*φ*=.634), followed by DAS-28 and SDAI. In contrast, in an Indian publication involving 200 patients, RAPID3 was best correlated with DAS28 (*ρ*=0.910), closely followed by CDAI (*ρ*=0.907). This study also reported that there was some disparity in the remission and low levels of disease's activity, since they indicate a lower agreement than high-to-moderate activity; while the agreement between DAS28 and RAPID3 for near remission in the Indian study was low (64%), our work revealed a high agreement (91.3%). The agreement with low activity in our study was poor (11.1%), even lower than the Indian study (55%).

Regarding CDAI, it was revealed that 4% of patients were in remission according to CDAI and 100% of those patients were in remission according to RAPID3; 15% of patients met the criteria for CDAI's low activity and 55% of those patients were in low activity according to RAPID3 [18]. In our study, we found 9.5% of patients on remission with CDAI, 73.9% of those were in remission according to RAPID3, 17.5% had low activity with CDAI, and 83.3% had low activity with RAPID3.

A study performed by Kim et al. in a Korean population of 400 patients also showed lower agreement percentages in the near remission and low activity category, 54% with DAS-28 and 52% with CDAI [19]. According to our data, we did find a high agreement between RAPID3 and DAS-28 for near remission. For low activity between RAPID3 and CDAI, these differences can be explained by the fact that the studies were conducted in different populations, where patients react differently towards emotions and pain; this could overestimate or underestimate RAPID3 scores. A study performed to determine the impact of the culture in RAPID3 scores found out that cultural issues played a huge role in the outcome. Given the fact that there is some disparity between different populations, investigators discovered that South America patients had the highest scores, followed by Caucasians, African-Americans, and lastly Asians which showed the lowest score [20]. However, our study results showed that RAPID3 could still be useful to determine near remission and low activity status in populations similar to ours.

Correlation values between SDAI and RAPID3 were the lowest of all the activity indexes that we measured (r.77, p < 0.001), but they still indicate a strong correlation. We found a low agreement with moderate (67%) and high (63%) activity of the disease. This also coincides with a study performed in Mexico with a population of 126 patients, where they found even a lower agreement in moderate activity to high activity (46%) [21].

One limitation of this study is that we did not include the social history of the patients, which would have been helpful since RAPID3 is a subjective measure that depends on the patient's mood, perceptions, and self -functionality; knowing certain aspects of the patient's lifestyle may be useful when it comes to RAPID3 results interpretation.

To our knowledge there are not any publications that validate RAPID3 as a good clinical measure in Ecuadorian population. So our research will provide useful data that will allow physicians to safely incorporate RAPID3 into their daily routines allowing them to improve the assessment and management of Ecuadorian patients with rheumatoid arthritis.

We conclude that on the daily clinical practice the physician can choose any measurement that suits the best with its situation, without affecting the therapeutic needs, or the monitoring of the evolution of the disease.

We still have to consider that RAPID3 also has limitations and is not trying to replace the other measurements that are made of more meticulous components such as the joint count, which is critical to measure disease activity in RA or the physicians' clinical perception. But it is still important to highlight the excellent correlation that RAPID3 had with the previous measurements, so it can be a very useful complement to these traditional scores.

RAPID3 is a clinical index that mainly puts emphasis on the patient's auto-evaluation of how the disease is affecting him/her in multiple contexts of life; it does not only focus on the abilities of the patient to perform daily activities, but it also prioritizes the pain. This is a benefit of the RAPID3 compared to other indexes, because at the clinical practice it is very useful for physicians so that they can determine the right time to switch medications, in order to provide a better management of the disease. In addition we have to take into account the fact that even if the patients are on low activity status, if the pain is severe enough, they will not be satisfied with the treatment and it will be beneficial to include medication to ameliorate pain like painkillers or anti-inflammatory drugs that will improve the quality of life in patients with rheumatoid arthritis.

Other advantage of RAPID3 is that it does not need laboratory tests, so compared with test like DAS-28 or SDAI, it is a less expensive option. RAPID3 could be used in low-socioeconomic-status patients that cannot afford laboratory analysis, and if no other clinical index can be performed, RAPID3 gives physicians the chance to still be able to monitor the patient's disease activity with a congruent index and offers them the right management.

RAPID3 is clear and simple, so it is easier for the patients to complete. It is a self-assessment questionnaire that can be filled at the doctor office waiting room and it will only take physicians 10 seconds to calculate and assess it, allowing them to have the necessary time to perform a good physical examination. We are quite certain that this questionnaire would also be useful to improve care in public health units that are usually overloaded with work and where time availability is indispensable.

## Figures and Tables

**Table 1:** Demographic and clinical information of studied population.

Characteristics	Patients (n=200) n (SD, %)
*Age (years)*	55.4 (13.5)
*Years with rheumatoid arthritis*	8.8 (7.0)
*Gender*	
Male	29 (14.5)
Female	171 (85.5)
*Serology*	
Seropositive	166 (83.0)
Seronegative	34 (17.0)
*Comorbidities*	
Arterial hypertension	55 (27.5)
Type 2 DM	29 (14.5)
Hyperlipidemia	30 (15)
Osteoporosis	84 (42.0)
BMI	27.9 (4.5)
*Treatment*	
Conventional-DMARDs	196 (98.0)
Biologics	72 (36.0)
Corticosteroids	165 (82.5)
Tofacitinib	16 (8.0)

Notes: Type 2 DM, type 2 diabetes mellitus; BMI, body mass index, DMARD, disease-modifying antirheumatic drugs.

**Table 2:** Mean scores of rheumatoid arthritis activity assessed by each method.

Characteristics	Patients (n=200) mean (SD)
*RAPID 3 (n, %)*	14.1 (8.2)
I (23, 11.5)	0.9 (1.0)
II (18, 9.0)	4.8 (0.8)
III (42, 21.0)	9.32 (1.7)
IV (117, 58.5)	19.9 (4.7)
*DAS 28 (n, %)*	4.9 (1.7)
I (28, 14.0)	2.0 (0.3)
II (6, 3.0)	2.9 (0.1)
III (67, 33.5)	4.3 (0.6)
IV (99, 49.5)	6.2 (0.9)
*CDAI (n, %)*	21.6 (15.4)
I (19, 9.5)	1.0 (1.0)
II (35, 17.5)	6.7 (2.3)
III (59, 29.5)	17.0 (3.5)
IV (87, 43.5)	35.2 (12.1)
*SDAI (n, %)*	23.7 (16.7)
I (21, 10.5)	1.6 (1.1)
II (27, 13.5)	7.0 (1.9)
III (74, 37.0)	19.0 (4.2)
IV (78, 39.0)	39.8 (13.6)

Notes: I, near remission; II, low activity; III, moderate activity; IV, high activity

**Table 3:** DAS 28, CDAI and SDAI categories within RAPID 3 categories.

	*RAPID 3*						
	I (NR) 23 (11.5)	II (LS) 18 (9.0)	III (MS) 42 (21.0)	IV (HS) 117 (58.5)	Chi-square p-value	Cramer's V	Total 200 (100)
*DAS 28 (n, %)*							
I (remission 0-2.6)	21 (91.3)	3 (16.7)	3 (7.1)	1 (0.9)	.000^a^	.590	28 (14.0)
II (low activity 2.61-3.2)	1 (4.3)	2 (11.1)	3 (7.1)	0 (0.0)			6 (3.0)
III (moderate activity 3.21-5.1)	1 (4.3)	12 (66.7)	30 (71.4)	24 (20.5)			67 (33.5)
IV (high activity >5.1)	0 (0.0)	1 (5.6)	6 (14.3)	92 (78.6)			99 (49.5)
*CDAI (n, %)*							
I (remission 0-2.8)	17 (73.9)	1 (5.6)	1 (2.4)	0 (0.0)	.000	.634	19 (9.5)
II (low activity 2.81-10.0)	5 (21.7)	15 (83.3)	12 (28.6)	3 (2.6)			35 (17.5)
III (moderate activity 10.1-22.0)	1 (4.3)	2 (11.1)	24 (57.1)	32 (27.4)			59 (29.5)
IV (high activity >22)	0 (0.0)	0 (0.0)	5 (11.9)	82 (70.1)			87 (43.5)
*SDAI (n, %)*							
I (remission 0-3.3)	17 (73.9)	3 (16.7)	1 (2.4)	0 (0.0)	.000	.583	21 (10.5)
II (low activity 3.31-11.0)	5 (21.7)	10 (55.6)	10 (23.8)	2 (1.7)			27 (13.5)
III (moderate activity 11.1-26.0)	1 (4.3)	4 (22.2)	28 (66.7)	41 (35.0)			74 (37.0)
IV (high activity >26)	0 (0.0)	1 (5.6)	3 (7.1)	74 (63.2)			78 (39.0)

Notes: All data are presented as frequencies and percentages. Chi-square tests for associations between RAPID 3 and DAS28, CDAI and SDAI are significant at .05 significance level. NR, near remission (0-0.3); LS, low severity (3.1-6.0); MS, moderate severity (6.1-12.0); HS, high severity (>12).

^a.^ Fisher exact test performed.

## Data Availability

The data used to support the findings of this study are available from the corresponding author upon request.
